# Diverse Secreted Effectors Are Required for *Salmonella* Persistence in a Mouse Infection Model

**DOI:** 10.1371/journal.pone.0070753

**Published:** 2013-08-12

**Authors:** Afshan S. Kidwai, Ivy Mushamiri, George S. Niemann, Roslyn N. Brown, Joshua N. Adkins, Fred Heffron

**Affiliations:** 1 Department of Molecular Microbiology and Immunology, Oregon Health & Science University, Portland, Oregon, United States of America; 2 Center for Bioproducts and Bioenergy, Washington State University, Richland, Washington, United States of America; 3 Biological Sciences Division, Pacific Northwest National Laboratory, Richland, Washington, United States of America; University of Osnabrueck, Germany

## Abstract

*Salmonella enterica* serovar Typhimurium causes typhoid-like disease in mice and is a model of typhoid fever in humans. One of the hallmarks of typhoid is persistence, the ability of the bacteria to survive in the host weeks after infection. Virulence factors called effectors facilitate this process by direct transfer to the cytoplasm of infected cells thereby subverting cellular processes. Secretion of effectors to the cell cytoplasm takes place through multiple routes, including two separate type III secretion (T3SS) apparati as well as outer membrane vesicles. The two T3SS are encoded on separate pathogenicity islands, SPI-1 and -2, with SPI-1 more strongly associated with the intestinal phase of infection, and SPI-2 with the systemic phase. Both T3SS are required for persistence, but the effectors required have not been systematically evaluated. In this study, mutations in 48 described effectors were tested for persistence. We replaced each effector with a specific DNA barcode sequence by allelic exchange and co-infected with a wild-type reference to calculate the ratio of wild-type parent to mutant at different times after infection. The competitive index (CI) was determined by quantitative PCR in which primers that correspond to the barcode were used for amplification. Mutations in all but seven effectors reduced persistence demonstrating that most effectors were required. One exception was CigR, a recently discovered effector that is widely conserved throughout enteric bacteria. Deletion of *cigR* increased lethality, suggesting that it may be an anti-virulence factor. The fact that almost all *Salmonella* effectors are required for persistence argues against redundant functions. This is different from effector repertoires in other intracellular pathogens such as *Legionella*.

## Introduction

*Salmonellae* are versatile and robust organisms capable of surviving in diverse nutrient environments and infecting a wide variety of animals. *Salmonella enterica* serovar Typhimurium (or *S.* Typhimurium) causes self-limiting gastroenteritis in humans and a typhoid-like disease in mice. Because *S. enterica* serovar Typhi causes typhoid fever in humans, *S.* Typhimurium infection of mice is used as a model to study typhoid fever. Typhoid fever can be both an acute and chronic disease followed by a carrier state in which *S*. Typhi is carried indefinitely and shed without symptoms [1], [2]. *Salmonella* becomes systemic either by invading and destroying the M cells of the intestinal epithelium thereby gaining access to the lymphatic system or by carriage directly to the bloodstream within CD18 positive cells such as macrophages, dendritic cells and neutrophils [3], [4]. Intraperitoneal (IP) infection of mice is commonly equated with systemic infection [5], [6], although there are clearly multiple steps in the process [7]. IP infection of specific strains of mice results in persistence and allows identification of genes required for resistance to the adaptive immune system and other factors. In contrast, infection of susceptible strains of mice, containing mutations in Nramp1, only identifies mutations more sensitive to innate immune components.

During the course of infection, *Salmonella* uses two type three secretion systems (T3SS) to aid infection [8], [9]. The secretion systems are encoded within *Salmonella* pathogenicity island-1 (SPI-1) and *Salmonella* pathogenicity island-2 (SPI-2). The proteins secreted by these two T3SS directly manipulate host cell functions. SPI-1 is important for gaining entry into the host cell, intestinal inflammation, and dissemination [10]. Once inside the host cell, bacteria reside within a modified lysosome called the *Salmonella* containing vacuole (SCV). SPI-2 effectors are required for systemic infection, cell-to-cell spread, and for resistance to some bacteriocidal factors but not intracellular replication per se [11]. Some effectors are secreted independent of the T3SS and are translocated to the host cell via outer membrane vesicles (OMV) while the secretion mechanism of a few proteins is not yet known [12]. Effector functions include altering the membrane and cytoskeleton of the host cell, manipulation of vesicular trafficking, inhibition of cell death pathways, and blocking innate and adaptive immunity. For SPI-1 T3SS effectors, there is ordered secretion [13] and this is likely to be true for other T3SS. Both SPI-1 and SPI-2 have been reported to play roles in persistent infections, but the effectors responsible have not been elucidated.

Previous genome-wide screens identified genes essential for persistent infections in mice by microarray screening of a library of transposon mutations following infection to identify genes that are lost [14]. These approaches used transposon insertion libraries, which may miss genes due to insertion specificity, identify false positives, or affect the expression of downstream genes. Here we describe our efforts to determine the essentiality of 48 *Salmonella* effectors in murine pathogenesis and to differentiate those that are required for persistence. Rather than relying upon a conventional competitive index (CI) assay, which uses phenotypic differences to differentiate wild-type and mutant strains, we used a qPCR (CI_qPCR_) method recently developed in our lab [15]. Using this approach, we evaluated the *Salmonella* effector repertoire en masse to establish each effector's contribution towards persistence at different times after infection.

## Results

To study the role of different effectors in persistence, we made non-polar deletions in each of the 48 effector genes. Using homologous sequence-mediated recombination [16], each mutant was tagged with a unique 24-nt barcode sequence [15] (Table 1). Amplification efficiency of each barcode sequence was determined beforehand and only the ones with similar cycle threshold values in qPCR were used [15]. For the wild-type control in these experiments, we substituted the pseudogene STM0314 with its own unique barcode. This replacement did not impact *Salmonella* virulence [15]. The CI_qPCR_ method drastically reduces the number of mice required for the study as up to 25 strains can be simultaneously infected. The number of mutants that can be compared in a study is thus no longer limited by the scarcity of selectable markers, as is the case for a conventional CI test. The results obtained by using the CI_qPCR_ method were shown to be comparable to those obtained using the conventional CI test [15].

**Table 1:** Barcode sequences used to replace effector genes.

Gene number	Gene symbol	Barcode sequence (5′ to 3′)
STM14_3481	*sipA (sspA)*	ACAATAGCTCTGACGCTTAGTCAC
STM14_3484	*sipB (sspB)*	ATTCTAATTATCAGCAGTGCAGTC
STM14_3483	*sipC (sspC)*	AATGCTGTTCTTGATCGTGATAGT
STM14_3482	*sipD (sspD)*	GTACGTGAAAGCGGTGTGCTTGGT
STM14_2557	*sopA*	ATAGCTATGATCCTTATCGGCAGT
STM14_1237	*sopB (sigD)*	ATTGGAATAATGATGAGTGCCAGG
STM14_3550	*sopD*	GTCACCAACAGTGTAAACGCAAGA
STM14_2244	*sopE2*	AGTATTGAAATTCACAAAAATCTG
STM14_3462	*avrA*	ATTGTTGTTGCTGTTAGTGCTATG
STM14_1197	*gtgE*	CTTAATGTCAATGCTAATAGCGGT
STM14_928	*slrP*	ATGAGAGGGAGTGGCGGAGGTCGA
STM14_5561	*spvC*	GTGGTTGCTCGGGATATTGGCAGT
STM14_5560	*spvD*	GTGATTATTGCAAGAGGGGTAGTG
STM14_3477	*sptP*	GGTATTAAGCGGATGCATACGAGC
STM14_4996	*sseK1*	AGTATTCACATGGTGGTTATGAGA
STM14_1483	*sspH1*	GGTCTAGTCATGGGAACGAGAAAC
STM14_1912	*steA*	GTTAGGAATGTGGTCAGTGTTATG
STM14_1970	*steB*	AGGGGCAGAGACGATGATGCGAGG
STM14_4534	*cigR*	CGTGTTACGCTGAGCACTGTTCGC
STM14_3164	*gogB*	CTGCTTGGTGTCAGTAGCGTGGCG
STM14_1166	*gtgA*	AATAGCCTGAGTGGCGATGGGGGT
STM14_1233	*pipB*	GGCATGGTTGTGGTGGTCAGTCGG
STM14_3350	*pipB2*	ATCATTAGTGATGTTGTTGTGAGG
STM14_1400	*sifA*	ATTATTACGGTGGCTCTTGCTGTT
STM14_1940	*sifB*	ATTGTTGGTAGGCAGGGTGTGAGG
STM14_1098	*sopD2*	ATCATTAATGCTCTGACCCTGAGC
STM14_1688	*spiC (ssaB)*	GTTGGGGTCGTGGTGATTGGCGCA
STM14_5562	*spvB*	GTTGTGAGGAGCGTCCATCGTGGT
STM14_3110	*sseB*	CGTATGCGTACGGATGGGGGGAGG
STM14_1696	*sseC*	CTGGTGATGAGCATGGGACGCAGG
STM14_1697	*sseD*	CGCAGTGGAGATGATGGTGTGGGT
STM14_1700	*sseF*	ATTATGAGAGTCCCAATTGTGATC
STM14_1701	*sseG*	ATACTGGGCACAGTCGTCGGTATT
STM14_1193	*srfH (sseI/gtgB)*	AAGGTTATTGACAGGAAGCGTGTG
STM14_1974	*sseJ*	AGGGTAATGGTCAGGGGTGTTGTG
STM14_2636	*sseK2*	AATAATGGGAGGAGCGGTGGTCTC
ST64B coliform bacteriophage	*sseK3*	ACTAACGTGGTTATCGACAGGCTG
STM14_2824	*sseL*	GTAATGAATAGTGCCAGGATCACG
STM14_2769	*sspH2*	CTCAGAAGTGTCAGAATGCTAAGT
STM14_2050	*steC*	CGTCTTGTTCTGAAGGCTATTAGT
STM14_2638	*steD*	ATTAGTGTGATGGCTGCTAACGGC
STM14_3166	*pagJ*	ATTGGTAAAAGGCATGTGGACAGC
STM14_2265	*pagK1*	ATTCCAATGCAAGGTGCAACCAGC
STM14_3167	*pagK2*	CAAAGTGCAATGAGAGTACTTGGC
STM14_1501	*pagC*	ACGCGCATTGTGGGTACAAGGGTA
STM14_1497	*pagD*	CGTATTGTTGTGGGAAGTCTCGTT
STM14_0421	*sssA*	GCTGGTATCATGATTCGCATGCTG
STM14_1785	*sssB*	GAAGTTTTTGTTCTTATGCTTGGT

Each mutant strain was mixed in equal proportions with a wild-type reference strain and co-infected by IP injection into female 5–6 week old 129SvJ mice at 10^4^ CFU in total. This strain of mice is now commonly used for persistence studies [14]. For IP infection there does not appear to be a bottleneck for access to systemic sites [17], [18] because a large number of targets were observed and most proved to be attenuated when subsequently tested. In contrast, each time mice are orally infected with a mixture of strains, a subset of only a few strains are recovered from the spleen and those strains are different upon repetition of the experiment [19], [20].

Groups of five mice were euthanized at days 2, 6, 9, and 14 post-infection. Previous persistence studies in mice recovered bacteria from both spleen and liver. However, we observed few differences between the two organs [15] and the spleen contains all WBCs in which *Salmonella* has been observed to replicate [21]. For that reason, we analyzed bacterial populations recovered from the spleen. Barcode sequences from each mutant were quantitatively measured by qPCR and compared to the CI value from the wild-type reference strain to establish a completive index (CI_qPCR_). For each effector we used an arbitrary five-fold cut-off to establish significant differences in the CI_qPCR_ values although the small standard deviation suggests that this is conservative. All mouse infections were performed at least twice for each time point. The following analysis is demarcated based upon previously published secretion pathways.

### Effectors reported to be translocated only by the SPI-1 T3SS

**Figure 1:**
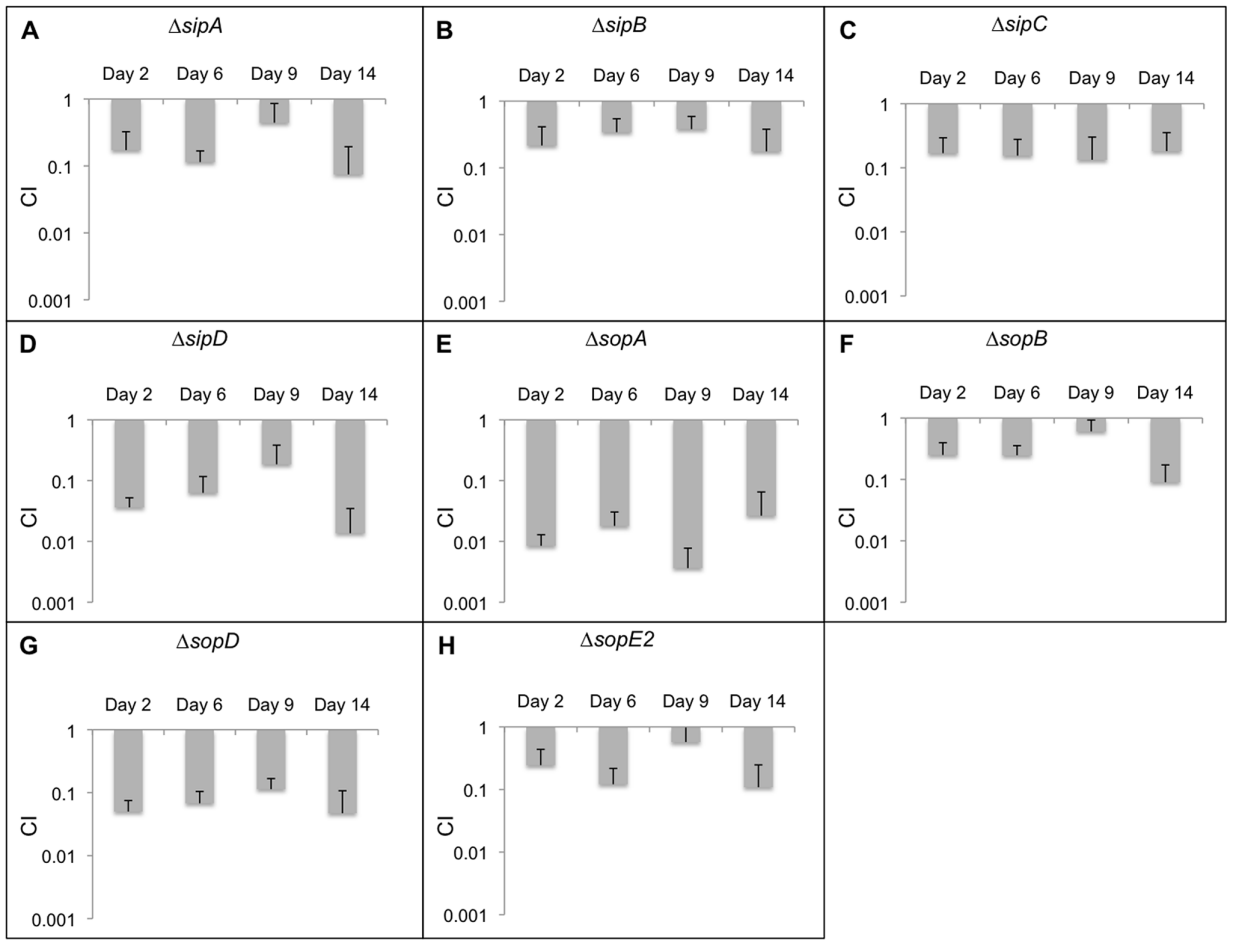
CI_qPCR_ analysis in 129SvJ mice for effectors secreted exclusively by SPI-1. 20 mice were infected with mutant strains as well as the wild-type control. Spleens from 5 mice were extracted at days 2, 6, 9, and 14 post-infection. The persistence levels of the various strains were determined using qPCR. The average CI_qPCR_ values are depicted here and the error bars indicate the standard deviation. Strains with CI_qPCR_ values greater than 1 demonstrate that these strains outcompeted the wild-type strains and strains with CI_qPCR_ values less than 1 indicate that they were outcompeted by the wild-type strain. Each panel from A to H represents CI_qPCR_ data for a mutant strain obtained at 2, 6, 9, and 14 days post-infection.

SPI-1 effectors are required for the invasion of epithelial cells and intestinal inflammation, which results in gastroenteritis. This secretion system has also been shown to be essential for persistence in mice [14] and this was true in our study as well. The CI_qPCR_ data for all SPI-1 effectors is depicted in Figure 1 and is summarized next.

During the course of our study, the Δ*sipA* strain was attenuated at all time points tested (Fig. 1A). SipA is produced and stored in the cytoplasm by *Salmonella* prior to cell invasion [22]. SipA injection activates the protein kinase C alpha (PKCα) signaling pathways and causes pro-inflammatory stimulation, which leads to the recruitment of professional phagocytes that carry *Salmonella* from the lumen of the intestine to the blood stream [21], [23]. SipA also inhibits actin depolymerization and increases cell invasion frequency [24], [25].

SipB and SipC insert into the plasma membrane and form a channel in the host plasma membrane that permits effector translocation but they also possess enzymatic activities. SipB associates with the proapoptotic protease caspase-1, and induces apoptosis of macrophages possibly permitting bacterial dissemination during infection [26]. *Salmonella* invasion of epithelial cells takes place via ruffling of the host cell membrane, which is caused by actin rearrangements [27]. SipA and SipC limit host membrane ruffling to the point of bacterial contact [28], [29]. Both the s*ipB* and *sipC* mutants were attenuated for persistence (Fig. 1B, C). Similarly, SipD forms the tip of the SPI-1 T3SS needle and regulates translocon assembly and effector translocation [30]. The Δ*sipD* strain showed between 10- and 100-fold decrease in persistence (Fig. 1D), reflecting its role in SPI-1 secretion. Unlike SipB and SipC, no other virulence function has been assigned to SipD.

The effector SopA showed the largest persistence defect of all SPI-effectors, about 100-fold by day 14 (Fig. 1E). SopA is a HECT E3 ubiquitin ligase mimic [31]–[33]. The strong phenotype of the *sopA* mutant suggests that this effector must be critical for persistence and the target of the ligase is of great interest. SopB is a phosphoinositide phosphatase that localizes to the host membrane early in infection and aids in the internalization of *Salmonella* [34]. SopB depletes the phagosomal membrane of negative charge, providing one of the mechanisms by which SCV-lysosome fusion is inhibited [35], [36]. Consequently, the Δ*sopB* strain also exhibited a persistence defect in mice (Fig. 1F). SopD is essential for optimal replication in mouse macrophages and has previously been shown to be required for systemic infection [37]. Likewise, we observed at least a 10-fold decrease in the number of Δ*sopD* bacteria compared to the parent even at the earliest time point (Fig. 1G). SopE2 is required for *Salmonella* invasion of epithelial cells and plays a role in the disruption of tight junction structure and function, which can lead to diarrhea when combined with the inflammatory response induced by other effectors [38]. In our study, Δ*sopE2* also had a persistence defect (Fig. 1H). We next examined effectors that can be secreted via either the SPI-1 or the SPI-2 encoded T3SS.

### Effectors reported to be translocated by either the SPI-1 or the SPI-2 T3SS

**Figure 2:**
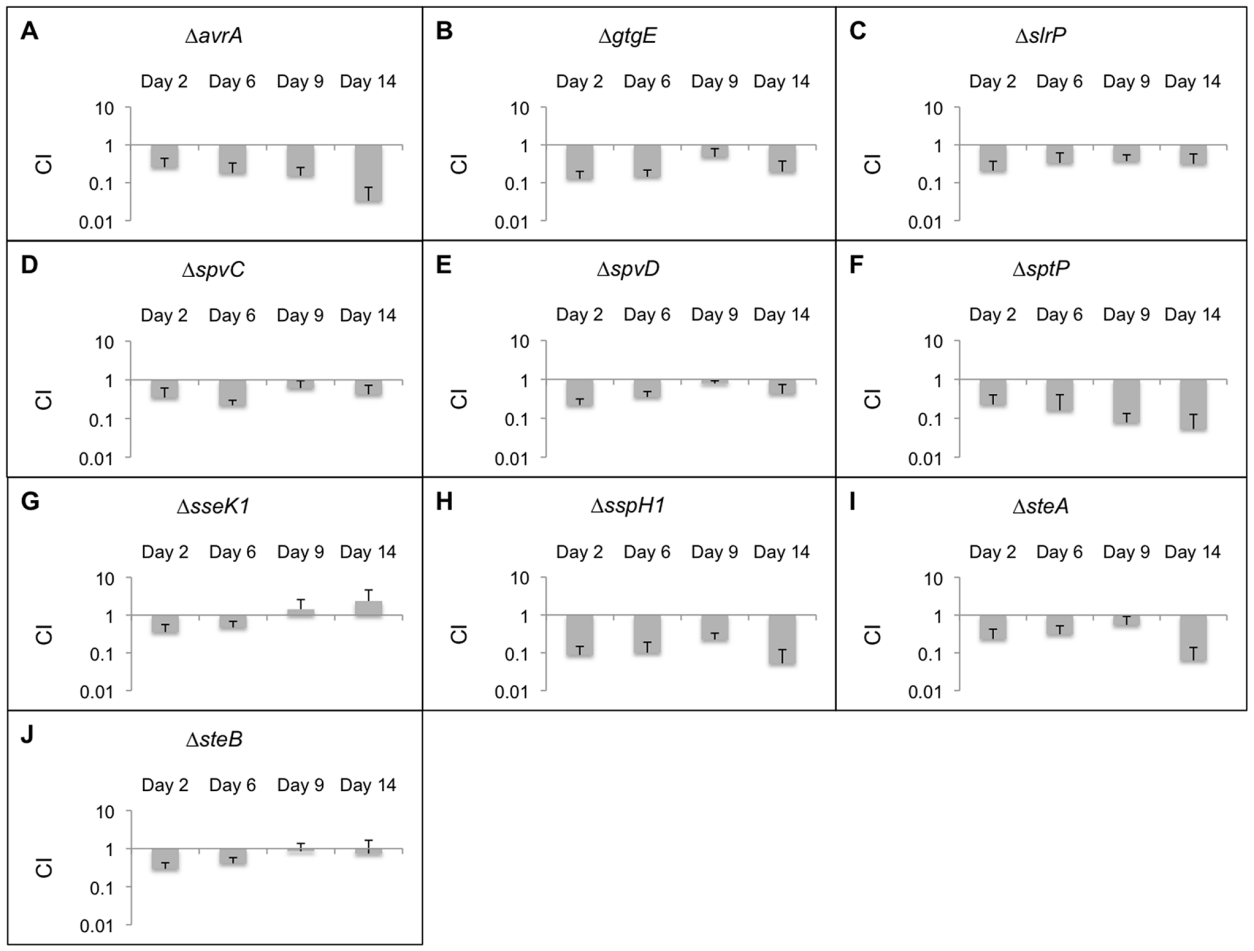
CI_qPCR_ analysis in 129SvJ mice for effectors secreted by both SPI-1 and SPI-2. 20 mice were infected with mutant strains as well as the wild-type control. Spleens from 5 mice were extracted at days 2, 6, 9, and 14 post-infection. The persistence levels of the various strains were determined using qPCR. The average CI_qPCR_ values are depicted here and the error bars indicate the standard deviation. Strains with CI_qPCR_ values greater than 1 demonstrate that these strains outcompeted the wild-type strains and strains with CI_qPCR_ values less than 1 indicate that they were outcompeted by the wild-type strain. Each panel from A to J represents CI_qPCR_ data for a mutant strain obtained at 2, 6, 9, and 14 days post-infection.

The CI_qPCR_ data for all effectors that can be secreted by either SPI-1 or SPI-2 is shown in Figure 2. The first strain, Δ*avrA*, showed a persistence defect of about 4-fold at early time points and was attenuated by more than 10-fold on day 14 (Fig. 2A). AvrA inhibits inflammation, represses apoptosis and innate immunity, and stabilizes tight junctions [39]–[41]. The Δ*gtgE* strain also showed a defect (Fig. 2B). GtgE is a unique lambdoid phage Gifsy-2 virulence factor with no significant homology discernable in the sequence databases [42]. GtgE alters vesicular traffic by cleaving Rab29 [43]. GtgE is not encoded in human adapted strains (*S*. Typhi and *S*. Paratyphi). SlrP confers host specificity, decreases thioredoxin activity, promotes cell death, and interferes with antigen presentation in dendritic cells [44]–[46]. According to our 5-fold cutoff, the Δ*slrP* strain was not significantly attenuated although it had reduced levels at all time points (Fig. 2C). SpvC has phosphothreonine lyase activity and inhibits MAP kinase signaling [47]. The Δ*spvC* strain did not exhibit a significant persistence defect as was observed for Δ*spvD*, which is in agreement with previous reports (Fig. 2D, E) [48].

SptP is a guanine activating protein (GAP). Its C-terminal domain is thought to prevent fusion with specific vesicles [49], [50]. We observed decreased persistence for the Δ*sptP* strain, which was pronounced (>10-fold) at days 9 and 14 post-infection (Fig. 2F). The *sseK1* deletion strain did not show a significant persistence phenotype (Fig. 2G). It has been demonstrated that after translocation, SseK1 localizes to the host cytosol, which is uncommon for *Salmonella* effectors [51]. SspH1 contains leucine-rich repeats that interact with mitogen activated protein kinase 1 [52]. SspH1 downregulates inflammatory responses, which may be a result of this interaction. The Δ*sspH1* strain showed about a 10-fold reduction when compared to the parent (Fig. 2H).

SteA is required for efficient mouse spleen colonization [53]. The *steA* deletion strain exhibited attenuation at all time points but the most significant difference was observed on day 14 post-infection (Fig. 2I). SteB is annotated as a putative dipicolinate reductase and was not significantly attenuated (Fig. 2J), confirming a previous study [53].

### Effectors reported to be translocated only by the SPI-2 T3SS

**Figure 3:**
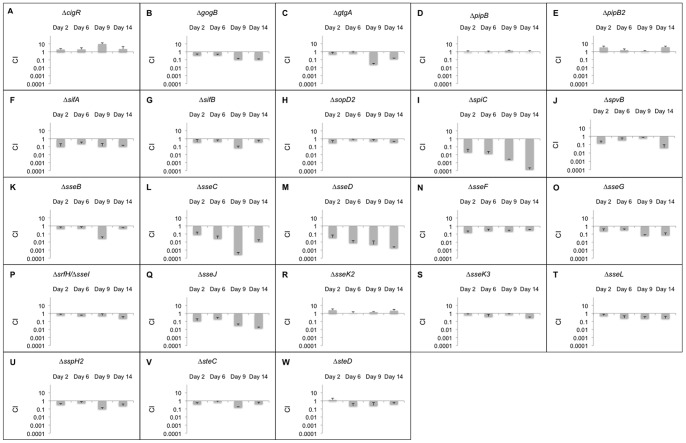
CI_qPCR_ analysis in 129SvJ mice for effectors dependent on SPI-2. 20 mice were infected with mutant strains as well as the wild-type control. Spleens from 5 mice were extracted at days 2, 6, 9, and 14 post-infection. The persistence levels of the various strains were determined using qPCR. The average CI_qPCR_ values are depicted here and the error bars indicate the standard deviation. Strains with CI_qPCR_ values greater than 1 demonstrate that these strains outcompeted the wild-type strains and strains with CI_qPCR_ values less than 1 indicate that they were outcompeted by the wild-type strain. Each panel from A to W represents CI_qPCR_ data for a mutant strain obtained at 2, 6, 9, and 14 days post-infection.

**Figure 4:**
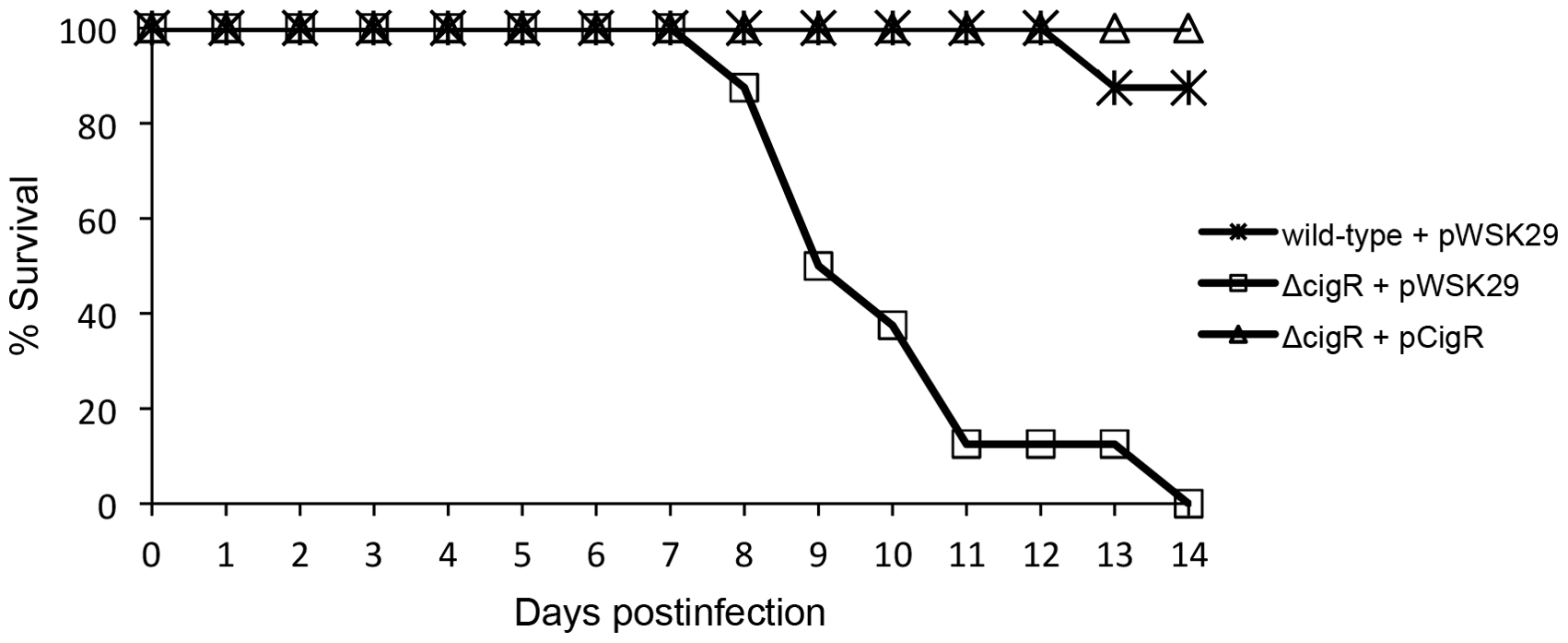
CigR shows anti-virulence activity. 129SvJ mice were infected IP with wild-type, Δ*cigR*, and a complemented derivative of this strain expressing cigR from a low coy plasmid. For each bacterial strain, five 129SvJ mice were infected at a dose of 10^4^ CFU/mouse and monitored for 14 days. The percentages of surviving mice are shown here for each strain. Differences in survival were statistically analyzed with a Kaplan-Meier test (p<0.0001). Mice infected with Δ*cigR* bacteria succumbed to infection, whereas mice infected with the wild-type or a complemented strain did not die.

The CI_qPCR_ data for all effectors that are dependent only on the SPI-2 T3SS is depicted in Figure 3. CigR is an effector that was discovered recently in our laboratory by proteomic analysis of culture supernatants [12]. In contrast to the other effectors we tested, persistence of the *cigR* mutant was elevated with respect to the wild-type strain at all time points, suggesting that this effector may be an anti-virulence factor. To confirm this observation, 129SvJ mice were infected IP with wild-type, Δ*cigR*, and a complemented derivative of this strain expressing cigR from a low coy plasmid. Mice infected with Δ*cigR* bacteria succumbed to infection, whereas mice infected with the wild-type or a complemented strain did not die (Figure 4). Thus, CigR functions as an anti-virulence factor.

A Δ*gogB* and a Δ*gtgA* deletion strain showed attenuation at 10 and 14 days post infection (Fig. 3B, C). GogB is encoded within the bacteriophage Gifsy-1. Likewise, GtgA is a bacteriophage encoded virulence determinant of unknown function [54], [55]. It is possible that these effectors are required for resistance to the adaptive immune system based on the fact that significant loss only occurred after day 7.

Both Δ*pipB* and Δ*pipB2* strains showed wild-type persistence levels (Fig. 3D, E). Neither PipB nor PipB2 has been reported to have a virulence phenotype [56], although they associate with detergent resistant lipid rafts on the SCV [57].

SifA is required for full virulence in mice. It plays an important role in maintaining SCV membrane integrity and in the formation of *Salmonella* induced filaments (SIF) [58], [59]. The Δ*sifA* strain showed a persistence defect of about 10-fold (Fig. 3F). SifB is also targeted to SCV and SIFs [60]. The *sifB* deletion strain displayed a small, but consistent persistence defect across all time points (Fig. 3G).

The Δ*sopD2* strain did not show a significant persistence defect compared to the wild-type strain (Fig. 3H). SopD2 aids in SIF formation, interferes with antigen presentation on dendritic cells, and is required for efficient replication in macrophages and mice [45] [37]. The discrepancy between the previously published results and our own may relate to the different strain of mouse being used (BALB/c Nramp1^−^ versus 129SvJ Nramp1^+^) [15]. This possibility has been observed before in which a mutation of *spvR* had a strong phenotype in Nramp1^−^ mice but none in congenic Nramp1^+^ [15].

The Δ*spiC* strain had a very strong persistence defect (Fig. 3I) equaled only by Δ*sseC* and Δ*sseD*. All are components of the secretion apparatus. SpiC was originally reported as an effector that interferes with vesicular trafficking, vacuole-associated actin polymerization (VAP), and SIF formation [61], [62]. Recent studies have demonstrated that SpiC forms a complex with SsaM and SsaL to regulate effector translocation [63]. A mutation of *spiC* is tantamount to a deletion of the SPI-2 T3SS although this does not rule out additional roles in virulence.

SpvB is essential for delayed host cell death by apoptosis following intracellular infection and inhibits VAP and SIF formation [64]–[66]. The Δ*spvB* strain showed more than 10-fold attenuation compared to wild-type but only at day 14 (Fig. 3J), perhaps suggesting a role in dissemination or resistance to adaptive immune components.

SseB, C and D form the needle tip of the SPI-2 T3SS and the translocon pore through the mammalian vesicle membrane [30], [67]. Deleting *sseB*, *sseC*, and *sseD* led to persistence defects following infection (Fig. 3K, 3L, 3M). SseF and SseG contribute to SIF formation along with SifA and are also important for positioning the SCV near the perinuclear/Golgi region [68], [69]. Deleting these two genes reduced persistence although the phenotype for *sseG* was stronger than *sseF* (Fig. 3N, O).

SrfH (SseI) promotes rapid septicemia minutes after oral infection, blocks dendritic cell motility, and reduces the inflammatory response [4], [70]. The *srfH* deletion strain showed attenuation at later times (Fig. 3P), which is consistent with previous reports [70]. SseJ is a cholesterol deacylase and also has acyltransferase activity [71], [72]. SseJ is required for intracellular replication in the host, and the Δ*sseJ* strain was attenuated for persistence (Fig. 3Q).

The *sseK2* and *K3* deletion strains were not significantly attenuated in our study (Fig. 3R, S). In BALB/c mice, deletion of *sseK2* did not have any effect on virulence [51]. Other researchers found a small effect of the *sseK* family on virulence but observed no effect by deleting *sseK3* alone [73]. The SseL effector inhibits apoptosis and downregulates the inflammatory response [74], [75]. However, it was not significantly attenuated in our study (i.e. <5-fold) (Fig. 3T). As there are multiple effectors already identified that inhibit innate immune signaling and inflammation, SseL may be redundant or simply not necessary in this infection model.

Several *Salmonella* effectors share sequence similarity within the first 120 amino acids (SlrP, SspH1, SspH2, SrfH (SseI), SseJ, SifA and SifB) [76]. SspH2 and SrfH differ by only two amino acids within the first 129 amino acids. This conserved region encodes an S-palmitolylation motif (GSGC) that begins 6 bp from the annotated N terminus and requires sequences encoded within the first 100 AA for palmitolyation. Palmitolylation is necessary for plasma membrane localization and activity (at least of SrfH). SspH2 is predicted to be a unique E3 ubiquitin ligase for which the target protein has not yet been identified. The *sspH2* deletion strain showed a persistence defect (Fig. 3U). SspH2 has been shown to be important for virulence in a calf model [77] and it interferes with antigen presentation in dendritic cells [45]. SteC has kinase activity that is required for actin remodeling in infected cells [78]. SteC has been previously shown to be important for virulence [53], but the *steC* deletion strain was only mildly attenuated for persistence in 129SvJ mice (Fig. 3V). In contrast, the *steD* deletion strain did not show a persistence defect (Fig. 3W), which is in agreement with a previous study [53].

### Effectors reported to be secreted independent of T3SS

**Figure 5:**
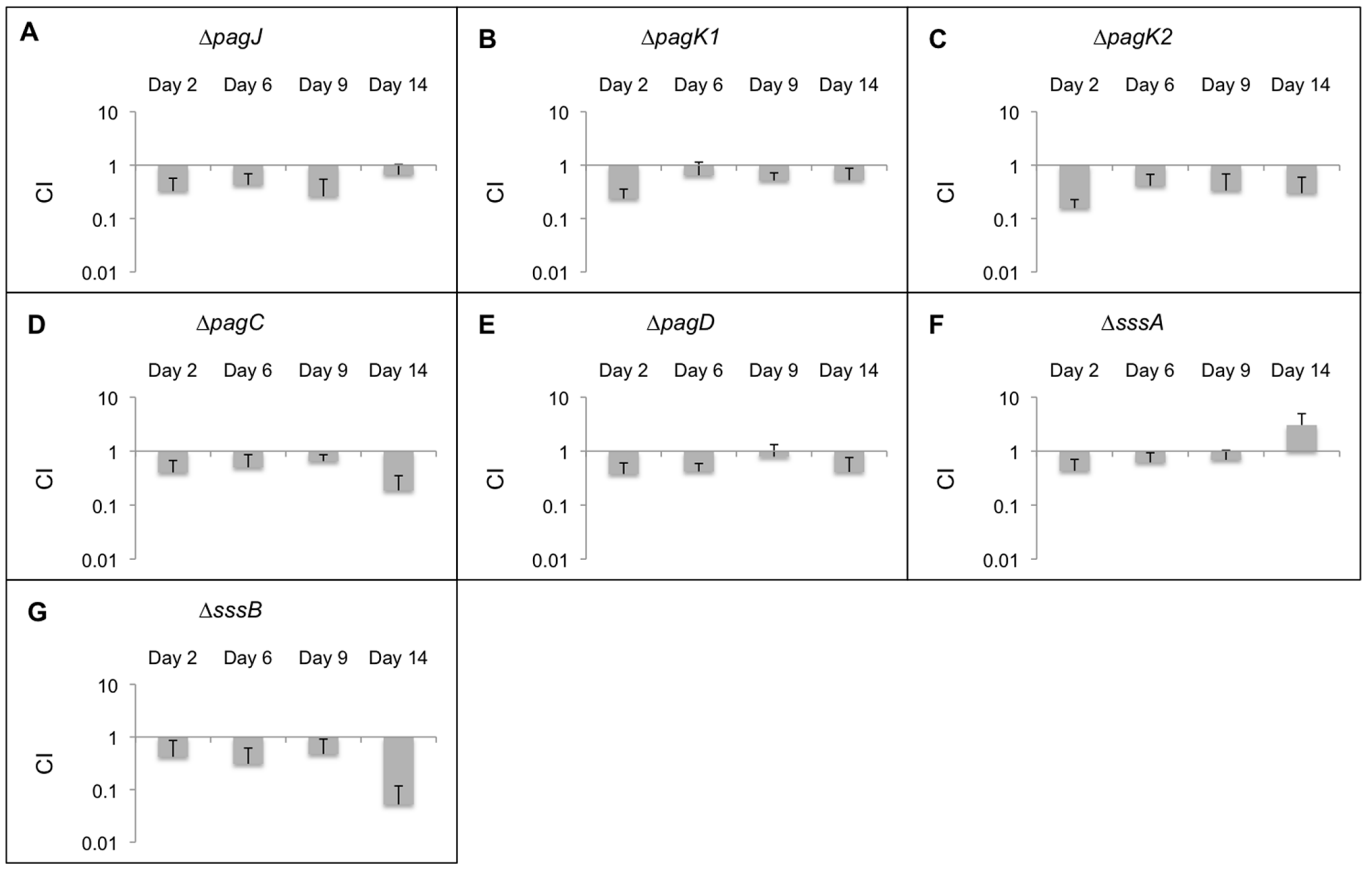
CI_qPCR_ analysis in 129SvJ mice for effectors independent of T3SS. 20 mice were infected with mutant strains as well as the wild-type control. Spleens from 5 mice were extracted at days 2, 6, 9, and 14 post-infection. The persistence levels of the various strains were determined using qPCR. The average CI_qPCR_ values are depicted here and the error bars indicate the standard deviation. Strains with CI_qPCR_ values greater than 1 demonstrate that these strains outcompeted the wild-type strains and strains with CI_qPCR_ values less than 1 indicate that they were outcompeted by the wild-type strain. Each panel from A to G represents CI_qPCR_ data for a mutant strain obtained at 2, 6, 9, and 14 days post-infection.

The CI_qPCR_ data for all effectors that are not secreted by T3SS is represented in Figure 5. PagJ, PagK1, and PagK2 are close homologs with unknown functions [79]. They are translocated into cells by outer membrane vesicles (OMV), and deletion of these genes showed a small persistence defect (Fig. 5A, 5B, 5C). Our results for PagK2 are consistent with a previous report [79]. The *pagC* deletion strain also showed attenuation in 129SvJ mice but only after 9 days (Fig. 5D). PagC is a membrane bound effector present in the periplasm, in OMV, or on the outside of the cell and helps *Salmonella* resist defensins, and potentially other microbiocidal host factors present in the phagolysome [80]. PagD may play a role in the survival of *Salmonella* within macrophages [81]. The *pagD* deletion strain was less persistent than the wild-type (Fig. 5E). Both PagC and PagD were identified in a secretome analysis of proteins secreted under conditions partially mimicking infection and may be secreted in OMV although this has not been directly demonstrated [12].

The secretion mechanisms for SssA and SssB/YdgH are unknown but may be OMV. The *sssA* deletion strain did not show significant attenuation compared to wild-type (Fig. 5F). The *sssB* deletion strain was attenuated compared to wild-type at later time points (Fig. 5G). SssB is secreted by SPI-2 but appears to also go out via OMV based on secretion that was partially dependent on T3SS. Proteins that are secreted via OMV have a secretion signal that directs them to the periplasm, which is observed in YdgH although not all periplasmic proteins are secreted [79].

## Discussion

In this study we evaluated the effect of deleting 48 *Salmonella* effectors on persistence, an important virulence attribute [6], [14]. By using a CI_qPCR_ method, we were able to mix multiple mutant strains with wild-type bacteria, thereby reducing the number of mice required. This approach yielded several intriguing observations. We found that all but seven effectors, SseK1, SteB, PipB, PipB2, SopD2, SseK2, and SseK3, contributed to persistence. Several of the mutant strains (*avrA*, *steA*, *pagC*, *spvB*, *sopB*, and *sssB*/*ydgH*) did not show a strong defect until two weeks after infection while others were lost within the first few days of infection. This suggests that they confer resistance to different aspects of host defense. Interestingly, we also discovered that the CigR effector functioned as an anti-virulence factor.

The CI_qPCR_ method has many of the advantages of conventional competitive index assay in that even small differences in virulence can be observed but it does not have the disadvantage of requiring a different mouse for each mutant tested. We have used a persistence model because, as observed previously, it is more sensitive than an acute infection model and reflects additional virulence attributes [6], [14]. In an acute infection model, the mice succumb before they have a chance to mount an adaptive immune response whereas in a persistence assay there are likely to be many other factors that contribute to bacterial survival. In fact, several of the mutant strains did not show a strong defect until two weeks after infection including *avrA*, *steA*, *pagC*, *spvB*, *sopB*, and *sssB* (*ydgH*).

### SPI-1 effectors

SPI-1 was previously shown to be required for persistence in a murine model [14]. We found that deletion of all SPI-1 effectors individually led to a persistence defect. SopA was notable in that its deletion resulted in a >100-fold difference when compared to wild-type. This was surprising when compared to mutations within the translocase proteins SipB, SipC and SipD, which did not have as strong a phenotype as a *sopA* mutant. It is possible that SopA can be secreted by an alternative mechanism similar to SipA, which can be secreted by the flagella. To test for this possibility we constructed CyaA' fusions to SopA, SopB, and SopE2 and tested each of them for translocation into J774 macrophages when expressed from a strong constitutive promoter. Under these conditions, each of these fusions could be translocated by the SPI-2 encoded T3SS (data not shown). The difference between this result and what others have reported for their SPI-1 dependent secretion may simply be that their expression was poor under the conditions they employed (late log in LB medium). In fact, an earlier study demonstrated that *Salmonella* is present in all WBCs of the spleen of infected C57/Bl6 mice, even T-cells, which are normally non-phagocytic and even in a strain missing a structural component of the SPI-1 T3SS. Thus, SPI-1 effectors may have an additional secretion pathway to explain *Salmonella* invasion of non-phagocytic cells.

### Effectors translocated by either SPI-1 or SPI-2

For effectors that can be secreted by either SPI-1 or SPI-2 T3SS, we observed decreased persistence for all mutant strains except *ΔsseK1* at later times post-infection suggesting that they were essential for persistence in the host. Of these genes, mutations in *avrA*, *sptP*, *sspH1*, and *steA* showed at least a 10-fold decrease relative to the parent after two weeks.

### SPI-2 effectors

Most strains with deletions of SPI-2 effectors showed attenuation compared to wild-type except for *cigR*, *pipB*, *pipB2*, *sopD2*, *sseK2*, and *sseK3*. Deletion of effectors GogB, GtgA, SifA, SpiC, SpvB, SseC, SseD, SseG, and SseJ showed at least a 10-fold loss at two weeks. Of these effectors, SpiC, SseC and SseD showed the strongest effect probably because they are structural as well. A dual role, both as a component of the secretion apparatus and as an effector is suggested for SpiC, whereas SseC and SseD are translocon components although they were observed to be secreted previously [12]. CigR was unique in that a deletion of this effector increased virulence. In 129SvJ mice following injection with 10^4^ bacteria, a mutant of *cigR* killed all the mice by day 14 but the complemented mutant and wild-type parent did not (Figure 4). The simplest explanation for this observation is that CigR is specifically recognized by the innate immune system similar to many T3SS effectors in plants [82]. This possibility is also suggested by the fact that it is one of the few conserved effectors present in many enterics and thus direct recognition by a component of the innate immune system seems more likely.

### Effectors secreted independent of T3SS

Of all the effectors secreted via OMV, only SssB (YdgH) had a strong persistence defect at two weeks post-infection. The fact that it is secreted by OMV was based on its identification in the secretome and the presence of a signal peptide expected to deliver it to the periplasm. However, there was no direct identification in secreted vesicles. Because OMV are diffusible, the persistence defect we observed may be partially complemented by other strains in a mixed infection and would require testing individual mutations. Previously no virulence defect was noted for a mutation in *sssB*, but this was assessed in Nramp1^−^ mice following an acute infection. SrfN has a structure related to SssB and is secreted by OMV based on direct identification. SrfN shows a small virulence defect. These effectors are part of a large family of conserved structurally related proteins without a known function (duf 1471) but present in many enteric bacteria.

### Complementation by co-infection

One question that arises when considering a large number of *Salmonella* mutants co-infecting a mouse is whether a factor produced by one mutant could complement a defect present in another mutant. For example, if the mutation is located in a diffusible factor such as a protein secreted in OMV, the other bacteria could provide the factor. These mutants will not be observed unless mutations are tested one by one. We (and others) have looked for such mutants unsuccessfully. For any genes that normally inhibit the adaptive immune response, the phenotype of the mutant in a mixed infection is less clear. Certainly if most bacteria are inhibiting T-cell replication and only a few do not, those that inhibit are more likely to win and thus the defect would not be observed. Both SrfH (SseI) and AvrA inhibit the adaptive immune response and yet are lost from a mixed infection. Surprisingly, the opposite effect has been noted i.e. mutations that are only observed during a mixed infection but when tested one by one have no phenotype [83].

### Persistence pathways

To our knowledge this is the first time that 48 *Salmonella* effectors have been studied en masse for a persistence phenotype. Surprisingly, the vast majority was required, suggesting that *Salmonella* effectors lack redundant functions. This observation contrasts starkly with other bacteria such as *Legionella* where effector deletion has no effect and is compensated by other effectors with similar activities [84]. Persistence may be a consequence of effector activities and is likely coordinated. *Salmonella* at systemic sites can re-seed the gut via the hepatic duct, but this may not be the only route. Identifying the effectors responsible for persistence, as we have done, is the first step in elucidating complex virulence pathways and how the bacteria responds to different environments within the host by expressing specific effectors.

## Materials and Methods

Mouse experiments were approved under the protocol of the Oregon Health & Science University Institutional Animal Care and Use Committee (OHSU IACUC; IS00001304: 2013) and performed in accordance with the guidelines for the Care and Use of Laboratory Animals of the National Institutes of Health to minimize animal suffering.

### Bacterial strains and plasmids

All *Salmonella* strains used in this study are *Salmonella enterica* serovar Typhimurium 14028 s and its isogenic derivatives. The mutant strains with deletions of different genes were generated using modified pKD13 (pKD13-mod) plasmids (pKD13; GenBank accession number AY048744), which were designed to replace target genes with 135-nucleotide (nt) barcode sequences following homologous recombination [15]. Briefly, linearized PCR products amplified from a pKD13-mod plasmid comprise a *kan* cassette in the middle and a 40-nt sequence at each terminus. The 40-nt termini are homologous to the target gene and facilitate homologous recombination at the appropriate chromosomal location [16]. PCR was used to confirm the substitution of the gene of interest by a *kan* cassette. Then the mutant allele was moved to a “clean” genetic background by using P22 transduction and the position of the *kan* cassette in the transductant was confirmed by PCR. The elimination of the *kan* cassette was carried out via site-specific recombination by expressing FLP recombinase in trans, resulting in in-frame, nonpolar deletions of target genes [16]. The deletions were confirmed by PCR as well as DNA sequencing. Table 1 depicts the unique sequences inserted in place of target genes. 48 effector genes were deleted for this study, and are catalogued in Table 1. For complementation experiments, the *cigR* gene plus 1,000 bp of upstream sequence encoding the promoter were PCR amplified from genomic DNA using primers 5′-ACTAGTGGATCCCCCGGGTTCTGCTGGAAAAAGATCTCATG-3′ and 5′-GCTTGATATCGAATTCTTAATCAAATACGCCATTAATAATCG-3′. The PCR product was cloned into pWSK29 cut with SmaI and EcoRI using the In-Fusion Cloning Kit (Clontech). The resulting plasmid was electroporated into the 14028 s Δ*cigR* background. Corresponding controls were established by electroporating pWSK29 into both 14028 s and Δ*cigR* backgrounds.

### CI_qPCR_ in mouse

A slight variation of the method described previously was used [15]. The mutant strains were divided into two groups for the infection study. SPI-2 effectors were infected along with the wild-type strain in one experiment and all the other effectors were infected with the wild-type control in a separate experiment. Each *Salmonella* strain containing a deleted gene as well as the reference strain was grown overnight in Luria-Bertani (LB) broth separately. On the following day, all the strains were washed three times with phosphate-buffered saline (PBS). The optical density at 600 nm was measured (OD_600_) and the strains (including the reference strain) were mixed equivalently and diluted to 10^5^ CFU/mL. Female 5- to 6-week old 129SvJ mice (Jackson Laboratory) were intraperitoneally injected with 100 µL of the mixed *Salmonella* strains at a final dose of 10^4^ CFU/mouse. The number of injected bacteria was confirmed by plating serial dilutions of the inoculum on LB agar plates and counting colonies on the following day. A portion of the inoculum, corresponding to 2×10^8^ CFU, was resuspended in distilled water and used in nested PCR for determination of *C_T_* values of all the strains in the input. The mice were euthanized on days 2, 6, 9, and 14 after infection in order to compare bacterial persistence between strains. Five mice were used for each time point. The spleens were extracted from the mice, homogenized, and plated on LB agar to isolate intracellular *Salmonellae*. The bacterial colonies from these plates were scraped and resuspended in PBS. A mixture of bacterial cells corresponding to 2×10^8^ CFU was resuspended in distilled water and used as the template for nested PCR using two outside primers that are common to all the mutant strains. Barcode DNAs amplified from nested PCRs of input and output samples served as template DNAs in the qPCR step. The efficiency of each barcode was calculated as stated previously [15]. The procedure used for nested PCR and qPCR as well as the calculations for CI_qPCR_ were carried out exactly as described previously [15].

### CigR complementation

129SvJ mice were infected IP with wild-type, Δ*cigR*, and a complemented derivative of this strain expressing CigR from a low coy plasmid. For each bacterial strain, five 129SvJ mice were infected at a dose of 10^4^ CFU/mouse and monitored for 14 days. Differences in survival were statistically analyzed with a Kaplan-Meier test.
